# Association between genetically predicted telomere length and facial skin aging in the UK Biobank: a Mendelian randomization study

**DOI:** 10.1007/s11357-020-00283-0

**Published:** 2020-10-08

**Authors:** Yiqiang Zhan, Sara Hägg

**Affiliations:** 1 German Center for Neurodegenerative Diseases, Ulm, Germany; 2 Institute of Environmental Medicine, Karolinska Institutet, Stockholm, Sweden; 3 Department of Medical Epidemiology and Biostatistics, Karolinska Institutet, Stockholm, Sweden

**Keywords:** Telomeres, Aging, Facial aging, Mendelian randomization, Genetics, Skin aging

## Abstract

Are shorter telomeres causal risk factors for facial aging on a large population level? To examine if longer, genetically predicted telomeres were causally associated with less facial aging using Mendelian randomization analysis. Two-sample Mendelian randomization methods were applied to the summary statistics of a genome-wide association study (GWAS) for self-reported facial aging from 417, 772 participants of the UK Biobank data. Twenty single-nucleotide polymorphisms (SNPs) that were of genome-wide significance were selected as instrumental variables for leukocyte telomere length. The main analyses were performed primarily using the random-effects inverse-variance weighted method and were complemented with the MR-Egger regression, weighted median, and weighted mode approaches. The intercept of MR-Egger regression was used to assess horizontal pleiotropy. Longer genetically predicted telomeres were associated with a lower likelihood of facial aging (*β* = − 0.02, 95% confidence interval: − 0.04, − 0.002). Comparable results were obtained using MR-Egger regression, weighted median, and weighted mode approaches. The intercept of MR-Egger regression was close to zero (0.002) that was not suggestive of horizontal pleiotropy. Our findings provided evidence to support a potential causal relationship between longer genetically predicted telomeres and less facial aging.

## Introduction

Telomeres are comprised of hexanucleotide DNA repeats and a complex structure of surrounding proteins at the end of chromosomes, protecting genetic information by maintaining the stability of chromosomes during cellular divisions [1]. Each time a cell divides, a small amount of telomeric DNA is lost because of the inability of the polymerase to fully elongate the ends of DNA. Consequently, telomeres are shorten with each cell division and are therefore recognized as a potential biological marker for cellular aging [2]. Telomere attrition over time results in critically short telomere lengths and leads to cellular senescence and apoptosis in normal cells. Skin cells, whose turnover cycle could be driven in part by telomeres replenishment, have a proliferative capacity that varies from a few days in newborns to a few weeks in adults [3, 4]. And facial skin cells are of particular interest. Maintaining a fast renewal of skin cells could slow down facial aging and make people look younger.

Different molecular mechanisms have been suggested to explain facial aging, where telomeres as a marker for biological aging have recently been gaining momentum [5]. For example, an in vitro experiment showed that skin cells with telomeres lengthened by a procedure that delivered a modified mRNA encoding *TERT* to cells were able to divide more times than untreated cells [6]. However, studies like this one need to be further investigated in large population-based analyses.

Therefore, in this study, we aimed to examine the potential causal association between leukocyte telomere length (TL), which was correlated with TL in skin cells (correlation coefficient *r* = 0.83) [7], and facial aging using a Mendelian randomization (MR) approach [8] in data collected from 417,772 participants of the UK Biobank [9].

## Materials and methods

### Instrumental variable selection

The European Network for Genetic and Genomic Epidemiology (ENGAGE) conducted a genome-wide association study (GWAS) for leukocyte TL in 78,592 individuals of European ancestry [10]. Mean leukocyte TL was measured in a mixed population of leukocytes, and measurements were conducted using an established quantitative polymerase chain reaction technique which expressed TL as a ratio of the telomere repeat number (T) to a single-copy gene (S) [11]. Leukocyte TL measurements were standardized either by using a calibrator sample or by quantifying against a standard curve. In total, 20 single-nucleotide polymorphisms (SNPs) at 17 genomic loci were independently associated with leukocyte TL at a level of genome-wide statistical significance (*P* < 5 × 10^−8^). In our study, we use these 20 SNPs as instrumental variables, and included proxy SNPs through LDlink if SNPs found to be palindromic (Table 1).

**Table 1:** SNPs selected as instrumental variables and their associations with leukocyte TL in ENGAGE consortium

SNP	Chromosome	Position (hg37)	Gene	Effect allele	Other allele	*β*	se(*β*)
rs2695242 proxy of rs3219104 *r*^*2*^ = 1	1	226594038	*RARP1*	G	T	− 0.039	0.006
rs55749605	3	101232093	*SENP7*	A	C	− 0.037	0.007
rs7643115 proxy of rs10936600 *r*^*2*^ = 1	3	169512241	*TERC*	A	G	− 0.086	0.006
rs2320615 proxy of rs4691895 *r*^*2*^ = 1	4	164069949	*NAF1*	G	A	0.055	0.006
rs13137667	4	71774347	*MOB1B*	C	T	0.077	0.014
rs2853677	5	1287194	*TERT*	A	G	− 0.064	0.005
rs7705526	5	1285974	*TERT*	A	C	0.082	0.006
rs805297 proxy of rs2736176 *r*^*2*^ = 1	6	31622606	*PRRC2A*	A	C	0.034	0.006
rs34991172	6	25480328	*CARMIL1*	G	T	− 0.061	0.010
rs59294613	7	124554267	*POT1*	A	C	− 0.041	0.005
rs9419958	10	105675946	*STN1 (OBFC1)*	C	T	− 0.064	0.007
rs228595	11	108105593	*ATM*	A	G	− 0.028	0.005
rs76891117 proxy of rs2302588 *r*^*2*^ = 1	14	73399837	*DCAF4*	G	A	0.048	0.008
rs7194734	16	82199980	*MPHOSPH6*	T	C	− 0.037	0.006
rs62053580	16	74680074	*RFWD3*	G	A	− 0.039	0.007
rs3785074	16	69406986	*TERF2*	G	A	0.035	0.006
rs8105767	19	22215441	*ZNF208*	G	A	0.039	0.005
rs71325459 proxy of rs34978822 *r*^*2*^ = 1	20	62268341	*RTEL1*	T	C	− 0.122	0.022
rs73624724	20	62436398	*RTEL1/ZBTB46*	C	T	0.051	0.007
rs75691080	20	62269750	*RTEL1/STMN3*	T	C	− 0.067	0.009

### Facial aging GWAS

In the UK Biobank, the facial aging domain was measured by the question (field code 1757)—*Do people say that you look younger than you are, older than you are, about your age, do not know, and prefer not to answer?* Analyses of this variable were performed in 417,772 British participants using a mixed linear model-based tool (*fastGWA*) that has treated facial aging as an ordinal categorical variable while controls for population stratification by principal components and for relatedness by a sparse genetic relationship matrix as well as age and sex [9]. These data were analyzed and made publicly available by the Complex Trait Genomics lab (http://fastgwa.info).

### Statistical analysis

The data on the instrumental variables and the facial aging GWAS were harmonized by the respective chromosomes and positions (human genome build 37). The random-effects inverse-variance weighted (IVW) method was used as the primary estimator for the MR analysis [12]. This method has a higher statistical power with the assumption that all SNPs are valid instrumental variables. The weighted median approach [13], MR-Egger regression [14], and weighted mode method [15] were used as complementary analysis. The weighted median approach yields consistent estimates when at least 50% of the weights in the analysis are from valid instrumental variables. The MR-Egger regression can adjust for directional pleiotropy but is of low power. The intercept of MR-Egger regression is used as a test for horizontal pleiotropy. The weighted mode approach is consistent when the largest number of similar individual-instrument causal effect estimates comes from valid instruments, even if most instruments are invalid. To examine if there was a reverse causation between TL and facial aging, we conducted an additional MR analysis as bi-directional MR analysis using 98 SNPs of genome-wide significance for facial aging as instrumental variables in UKB. Then, we harmonized their respective effect sizes and standard errors for these SNPs in ENGAGE TL GWAS. All statistical analyses were conducted in R 3.6 and *TwoSampleMR* package [16].

The present study only used publicly available summary-level statistics. No individual-level data was analyzed. Ethical approval is therefore not required.

## Results

**Fig. 1:**
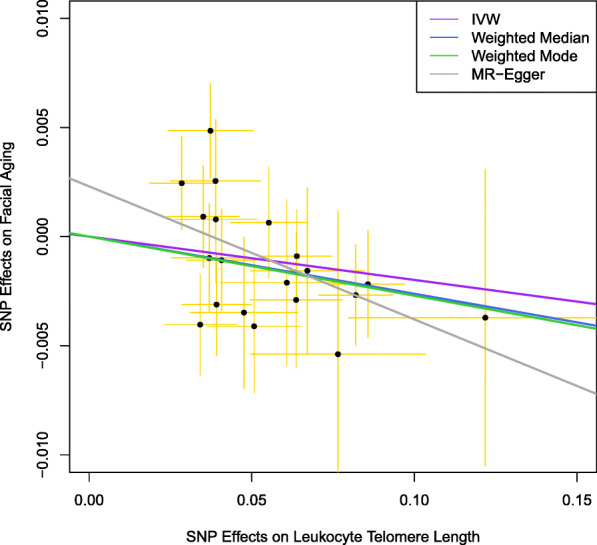
Facial skin aging. Scatter plot for the effects of SNPs on leukocyte telomere Length and facial aging. IVW, inverse-variance weighted; MR, Mendelian randomization. The horizontal axis represents the effects of each genetic variant on leukocyte telomere length, and the vertical axis denotes the effects of each genetic variant on facial aging. The orange lines around the solid black points are the corresponding confidence intervals for the effects. The slopes of solid lines represent the estimates from IVW, weighted median, weighted mode, and MR-Egger regression analyses

Table 1 describes 20 genetic variants as instrumental variables and their associations with leukocyte TL. Six palindromic SNPs were replaced by their corresponding proxy SNPs (*r*^*2*^ = 1). The scatter plot for the effects of these SNPs on leukocyte TL and facial aging is shown in Fig. 1.

The MR analysis shows that longer genetically predicted leukocyte TL was associated with a lower likelihood of facial aging (*β* = − 0.02, 95% confidence interval [CI]: − 0.04, − 0.002) using the IVW method (Table 2). Similar results were obtained through MR-Egger regression (*β* = − 0.06, 95% CI: − 0.12, − 0.002), weighted median (*β* = − 0.03, 95% CI: − 0.05, − 0.007), and weighted mode approaches (*β* = − 0.03, 95% CI: − 0.05, − 0.007). These estimates were also plotted in Fig. 1.

**Table 2:** Association between telomere length and facial aging in the UK Biobank

Methods	*β*	95% CI
Inverse variance weighted	− 0.02	− 0.04, − 0.0002
MR-Egger regression	− 0.06	− 0.12, − 0.002
Weighted median	− 0.03	− 0.05, − 0.007
Weighted mode	− 0.03	− 0.05, − 0.007

*MR* Mendelian randomization

**Fig. 2:**
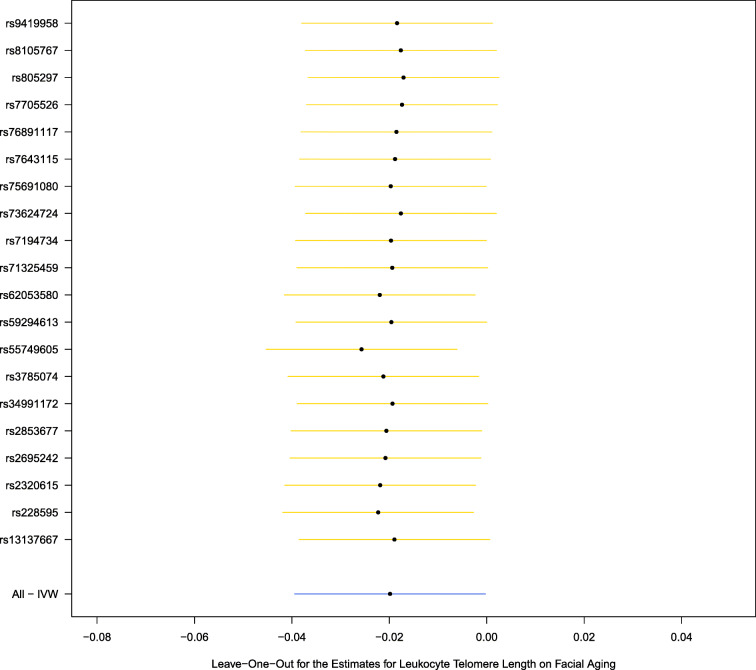
Facial skin aging. Leave-one-out analysis for the estimates for leukocyte telomere length on facial aging. IVW, inverse-variance weighted

**Fig. 3:**
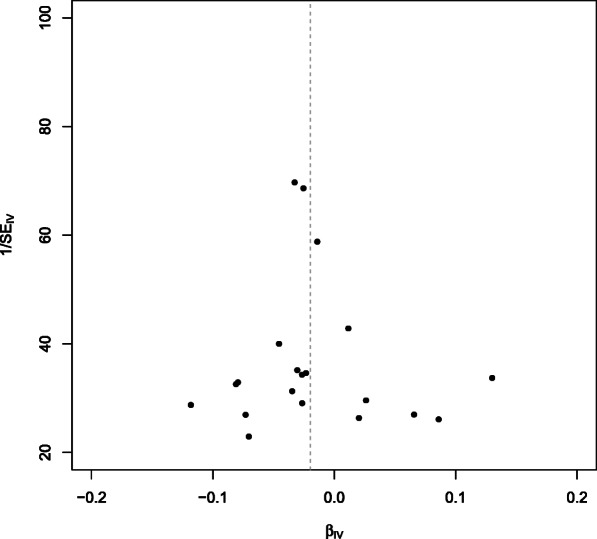
Facial skin aging. Funnel plot for the SNPs. IV, instrumental variable

We additionally performed a leave-one-out analysis, which yielded comparable results and did not find noticeable effects of any single SNP that could dominate the results (Fig. 2). The funnel plot did not imply that there were heterogeneous SNPs (Fig. 3). We did not find evidence for directional pleiotropy from the MR-Egger regression (intercept: 0.002, *P* = 0.16).

Additional bi-directional MR analysis was performed by using 98 SNPs that were of genome-wide significance for facial aging as instrumental variables. We did not find a significant effect of genetically predicted facial aging on leukocyte TL (*β* = − 0.08, 95% CI: − 0.22, 0.05).

## Discussion

In the present study, for the first time, we examined the association of genetically predicted leukocyte TL and facial aging in a large population-based cohort, the UK Biobank, using the MR method. By leveraging several MR estimation approaches, we found that longer genetically predicted leukocyte TL was associated with a lower likelihood of facial aging. Our study, corroborating previous experimental studies of skin cells, provides further evidence to support a causal role of leukocyte TL in facial aging.

To the best of our knowledge, no studies have been published to investigate the role of TL in facial aging using population-based cohorts until now. A previous study examined TL in cells of the sun-protected and sun-exposed skins [17]. Further studies employed ultraviolet-exposed skin cells to study the roles of TL in photoaging [18–21]. These studies, taken together, were suggestive of the functions of TL in skin aging at the cellular level [5, 22]. Further evidence at the population level, however, is lacking. The limited number of studies on this topic at the population level is partly due to the difficulties in facial aging measurements. For example, although the three-dimensional human facial morphology assessment instruments [23], among others [24], can offer objective and comprehensive features for facial aging, they have not been widely available for aging researchers because of infeasibility and high cost.

To overcome the challenges in facial aging measurement and to make the best use of questionnaire-based instruments, a subjective assessment of facial aging could be valuable in this regard measurement [25–27]. In this study, we examined the role of TL by taking advantage of questionnaire-based measurement for facial aging in the UK Biobank. An advantage of this type of data is that the large sample size in the UK Biobank could outweigh the concerns in measurement error for questionnaire-based facial aging assessment. Indeed, we obtained consistent and significant estimates by using various MR approaches in this study. Further studies are warranted to explore the biological mechanisms of TL in facial aging.

Despite the advantages of the large sample size, our study is prone to several limitations. First, the MR assumptions, particularly the no-pleiotropy assumption, must be satisfied in order to yield a valid estimate. The intercept of MR-Egger regression analysis approaches zero suggesting no strong evidence for directional pleiotropy. Second, the MR methods used in this study also assume a linear relationship between TL and facial aging. We cannot examine further if there was a non-linear relationship. Third, as facial aging GWAS was analyzed using mixed linear models, the effect sizes cannot be interpreted using a more intuitive way. The results could rather be used for testing purposes. Fourth, as alluded above, the facial aging was measured using a single question in the UK Biobank, which could lead to measurement error. Future studies are encouraged in order to develop a more detailed questionnaire-based instrument to measure diverse dimensions of facial aging. Fifth, in this study, facial aging was assessed only using qualitative data and not objectively assessed using skin turgor [28] or facial wrinkles per area of skin [29]. Lastly, TL was measured in leukocyte rather than skin cells. However, a previous study demonstrated that the correlation of TL in leukocyte and skin cells was high (correlation coefficient *r* = 0.83) [7], and a recent study found that TL varies by tissue type but is generally correlated among tissue types [30].

In summary, our study provided novel evidence to support a causal role of genetically predicted leukocyte TL in facial aging. Further studies are warranted to explore the biological mechanisms of TL in facial aging.

## Data Availability

The data are publicly available.
